# GENDER DIFFERENCES IN PSYCHOLOGICAL OUTCOMES OF DEMENTIA CAREGIVERS

**DOI:** 10.1093/geroni/igad104.2221

**Published:** 2023-12-21

**Authors:** Elena Roden, Francesca Falzarano

**Affiliations:** Fordham University, New York City, New York, United States; Weill Cornell Medicine, New York City, New York, United States

## Abstract

Substantial research has been conducted on the high prevalence of anxiety and depression among dementia family caregivers. Stressors, such as financial hardships related to costs of care and feelings of role captivity, can exacerbate adverse mental health outcomes. However, there is limited literature focused specifically on gender differences in the caregiving stress process. Using data from a mixed-methods study of 46 dementia family caregivers between the ages of 23-96, the purpose of this study was to examine gender differences in self-reported caregiving stressors (e.g., cost of care, care intensity) and psychosocial outcomes including depression and anxiety. Results showed that compared to females, male caregivers reported greater costs of care, greater feelings of role captivity, higher frequency of assistance with medical/nursing tasks (p=.01) and activities of daily living (ps range from .01-.04). Male caregivers also reported significantly greater distress related to care tasks (p=.04), as well as higher levels of depression (p=.02) and anxiety (p=.05). These findings provide insight into how the informal caregiving experience can vary by caregiver gender and potential factors contributing to these differential outcomes. The data establishes the importance of considering demographic characteristics in providing needed resources to dementia family caregivers.

